# Case Report: Leptomeningeal involvement in chronic lymphocytic leukemia patients with central nervous system infection: extramedullary manifestation or transmigration of leukocytes—how to treat this type of CLL?

**DOI:** 10.3389/fonc.2026.1788628

**Published:** 2026-05-05

**Authors:** Tianjiao Zhang, Annette Schuller, Yi Li, Regina Herbst, Korinna Jöhrens, Nicole Schleicher, Ronny Maurer, Mathias Hänel

**Affiliations:** 1 Department of Internal Medicine III, Klinikum Chemnitz gGmbH, Chemnitz, Germany; 2 Center for Diagnostic, Klinikum Chemnitz gGmbH, Chemnitz, Germany; 3 Haematology department, Sheffield Teaching Hospitals National Health Service (NHS) foundation, Sheffield, United Kingdom; 4 Institute for Pathology, Klinikum Chemnitz gGmbH, Chemnitz, Germany; 5 Oncological Group Practice, Chemnitz, Germany

**Keywords:** BTKi, CLL, CNS infection, leptomeningeal involvement, neuroborreliosis, Zanubrutinib

## Abstract

Central nervous system (CNS) involvement by CLL is rare. We report a non-treated CLL patient with low-risk factors, but showed leptomeningeal involvement during/after neuroborreliosis and EBV meningitis. An 80-year-old woman with CLL in Binet stage A showed neurological syndromes and increased leukocytosis. The initial cerebrospinal fluid (CSF) revealed EBV-meningitis and neuroborreliosis, but no evidences for CNS involvement of CLL by flow cytometry and morphological/immunochemical assays. After antimicrobial treatment, neurological syndrome were significant improved. However, leukemic meningeal involvement was proved in following CSF analyses. Treatment with intrathecal chemotherapy and Bruton’s tyrosine kinase inhibitor (BTKi) Zanubrutinib were performed. At the follow-up after 12 months, a complete remission was observed. Considering the case of our patient and previous reports, the question arises whether the detection of leukemic cells in the CSF of CLL patients with/after infectious meningitis (especially in neuroborreliosis) is an extramedullary manifestation or only a transmigration of clonal lymphocytes caused by the inflammation of the blood-brain barrier. The prophylaxis of CNS infections should be more seriously, since it may be associated with CLL leptomeningeal involvement. The need for an individualized therapy is certain, our case confirmed the efficacy of Zanubrutinib.

## Introduction

Chronic lymphocytic leukemia/lymphoma (CLL) is the most common hematological malignancy in adults. However, central nervous system (CNS) involvement by this indolent non-hodgkin’s lymphoma (NHL) is rare. Mayo Clinic reported a cohort involving 4174 patients with CLL, in which the incidence of CNS involvement was only 0.4% (1). A systemic analysis of case reported between 1975 and 2012 showed that extramedullar CLL affecting CNS has the worst prognosis (2). However, diagnosing CLL with leptomeningeal involvement can be challenging. CNS infections, such as neuroborreliosis and bacterial/viral meningitis, frequently present with similar or even identical neurological symptoms to meningeosis leukaemica (3). Furthermore, the link between such infectious lesion and disease progression with CNS manifestations remains unclear.

Here we report a patient with asymptomatic low-risk CLL (Binet stage A) who developed meningeosis of pre-existing CLL following neuroborreliosis and EBV meningitis.

## Case presentation

An 80-year-old woman was referred with leukocytosis, hyponatremia, hypokalemia, and increasing confusion, dizziness, ataxia and slowness. B-CLL was initially diagnosed in Binet stage A and only a wait-and-see approach was adopted. At the time of admission to our clinic, the patient was in a poor general and nutritional condition (ECOG 2). There was no peripheral lymphadenopathy, splenomegaly or hepatomegaly. Clinically, she had no strikingly cranial nerve abnormalities, but was apparently disoriented and slow in response. Laboratory tests revealed a significant leukocytosis at 153 Gpt/l, with a lymphocyte count of 137 Gpt/l. Hemoglobin, platelets, C-reactive protein (CRP) and Procalcitonin (PCT) were within normal limits. We performed CT scan of head and abdominal/lymph node ultrasound to exclude acute intracranial pathology and systemic progression of CLL. A total of nine lumbar punctures were performed during the hospitalization. The first sample of cerebrospinal fluid (CSF) was taken after two doses of empiric antimicrobial treatment and revealed pleiocytosis at 594 Mpt/l, in which was T-cell dominant, CD5+ B-cells at about 4%. Besides, with high lactate level at 4.1 mmol/l, protein level at 2506 mg/l and Epstein-Barr virus (EBV) Copies at 2652 IE/ml. The initial diagnosis was therefore viral meningitis.

With fever and increasing confusion, we initially started empiric treatment with acyclovir and meropenem (4). For EBV meningitis rituximab and immunoglobulins were administered, which resulted in subsequent EBV negativity in CSF samples from the 5. lumbar punction. The patient’s medical history indicated a tick bite about four weeks earlier. Unfortunately, no relevant tests were performed during the initial lumbar puncture examination. The suspected neuroborreliosis was confirmed by IgM positivity of Borrelia burgdorferi in CSF samples (titer increasing in the 2. and 3. lumbar punctures) and also in serum samples (slightly lower level than in CSF). The patient was then given ceftriaxone (5) for two weeks, which led to a significant neurological improvement.

**Figure 1:**
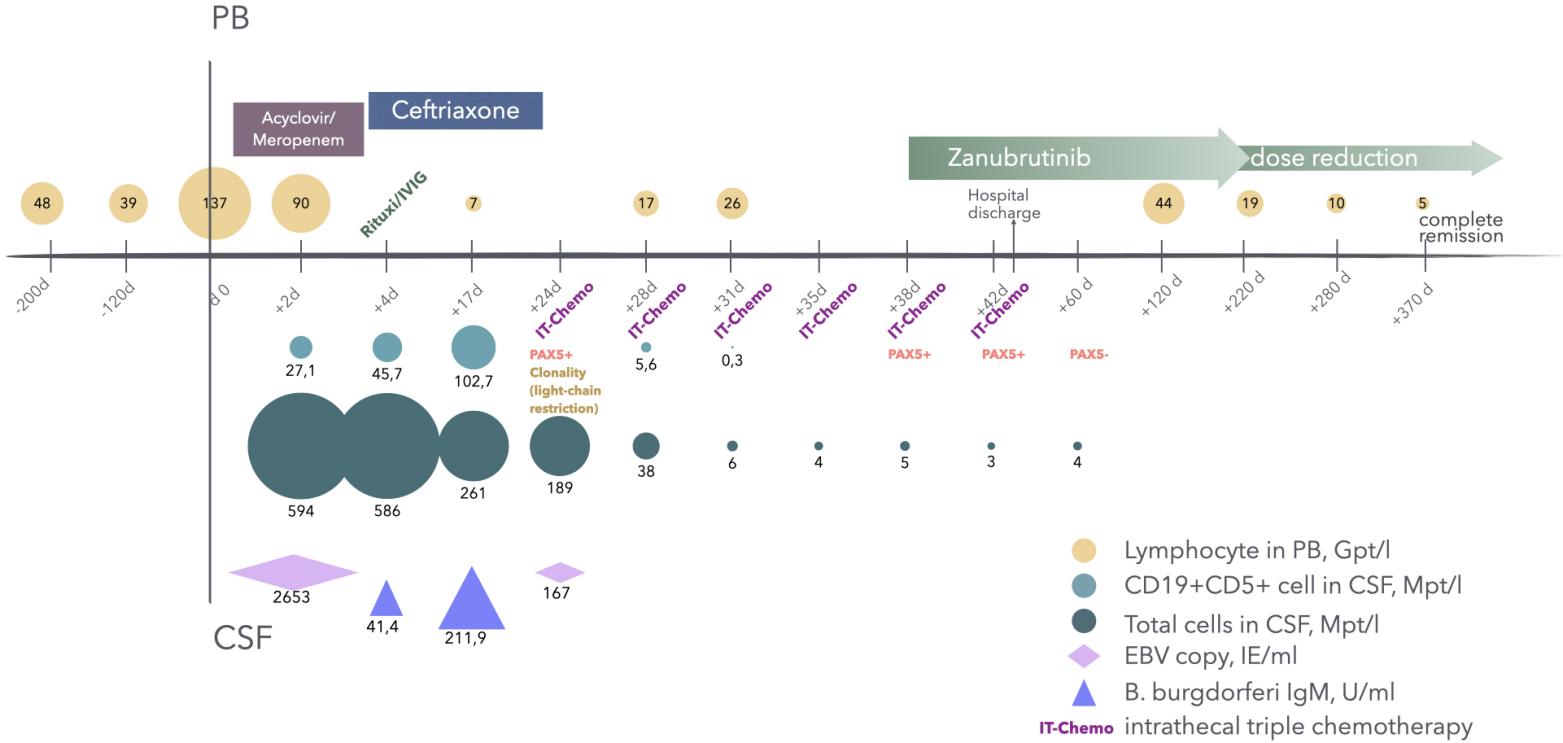
Time line around admission. Above the X-axis: lymphocyte counts in peripheral blood (PB) and treatment milestones. Under the X-axis: total cell counts, CD19+CD5+ clonal B cell counts, results of infectious serological tests and pathological results in cerebral spinal fluid (CSF), intrathecal chemotherapies..

**Figure 2:**
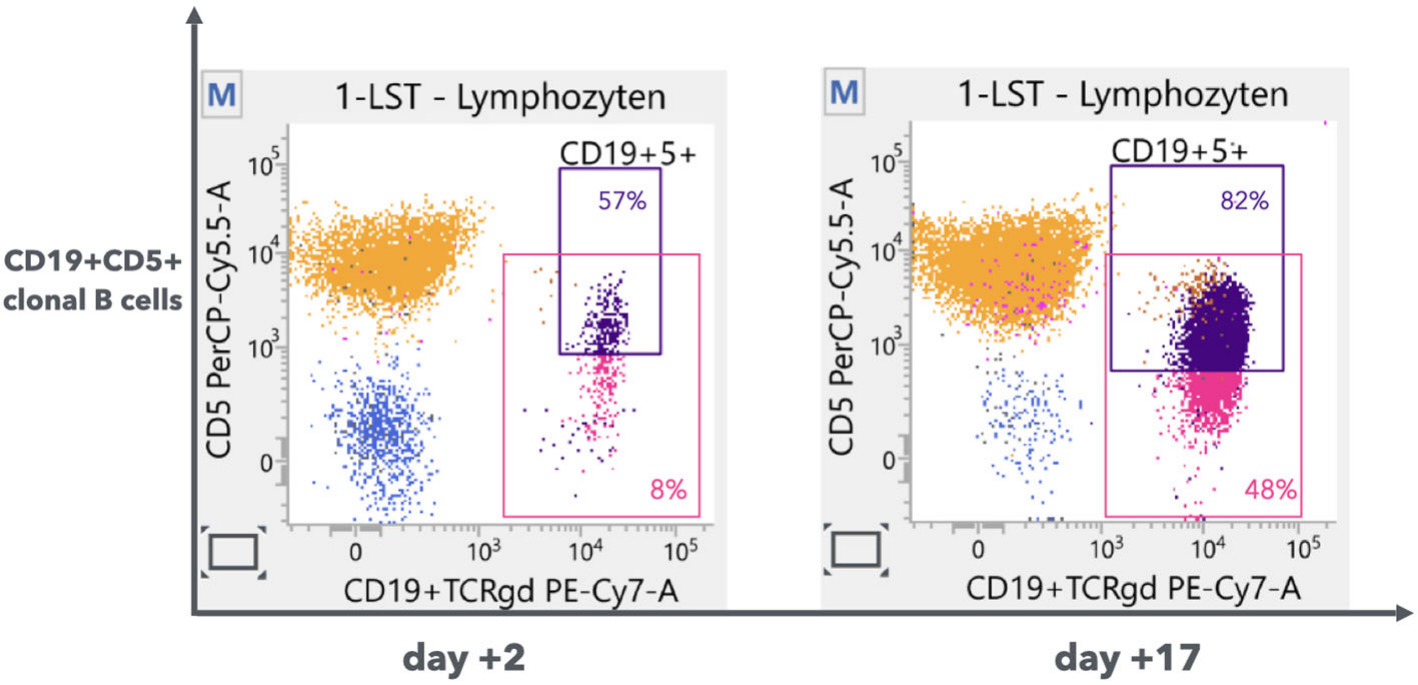
Immunophenotype of cells in CSF. Flowcytometry analysis of lymphocytes in CSF on d+2 (left) and d+17 (right) from admission. CD19+ B cells (pink gating: percentage of total lymphocytes) were significantly increased. Moreover, increased cells were mainly CD19+CD5+ clonal cells (purple gating: percentage of B cells).

Given the pre-existing CLL, CNS involvement was initially also under consideration, but flow cytometry and cellular immunochemistry of the initial CSF revealed no evidence of meningeosis leukaemica despite a significantly increased cell count in the CSF (**Figure 1**). With antimicrobial treatment, the neurological symptoms improved significantly and the total cell count in CSF from the 3. lumbar puncture decreased. In contrast, however, the CSF samples from 2. lumbar puncture showed an increased cell count of clonal CD19+ B lymphocytes with co-expression of CD5 (**Figure 1** and **2**), cells morphologically/immunochemically suspicious for leptomeningeal involvement of CLL (6). Besides, clonality of B lymphocytes in CSF was also confirmed through light-chain restriction in the 4. lumbar puncture. Cytological examination and microscopic evaluation were performed at each lumbar puncture. The CSF contamination related to puncture was ruled out since very few or no red blood cells were detected in CSF. Except for newly CNS involvement, other overall assessments indicated stable CLL and low risk profiling. Cytogenetic/molecular analysis revealed 13q14.3 deletion, mutated IGHV and TP53 wild type. A CT scan of the neck, chest, abdomen and pelvis ruled out splenomegaly, hepatomegaly and lymphadenopathy. The peripheral blood B-lymphocyte count was decreased to 7 Gpt/l, B-symptoms were not present.

Intrathecal chemotherapy with cytarabine, methotrexate and dexamethasone reduced the cell counts in the CSF samples from 261 Mpt/l to 5 Mpt/l within two weeks, but CSF cellular chemistry and immunphenotyping suggested ongoing infiltration of leukemia cells with persistent positive PAX5 (**Figure 1**, from d+24 to d+38), which is not specific to CLL but controls early B-cell development at transition to pro-B-cell stage and is expressed in most B lymphocyte malignancies (7). Bruton’s tyrosine kinase inhibitor (BTKi) with Zanubrutinib was started. The last lumbar puncture before hospital discharge showed total cell counts of 3 Mpt/l in CSF with highly expression of PAX5. Although clonal assay of B lymphocyte was not performed using this CSF, it still highly indicates ongoing leptomeningeal involvement. In view of the low cell count and the absence of neurological symptoms, the intrathecal therapy was suspended while Zanubrutinib was continued. On discharge, she was oriented in person, place and event, while the persistent disorientation in time could be an indication of an additional dementia development.

A prompt follow-up including lumbar puncture took place in 1 week after discharge (2 months after diagnose, 3 weeks after Zanubrutinib start). This CSF showed negative PAX5. At the last follow-up (more than 12 months after discharge), she continued to take Zanubrutinib with a reduced dose(80 mg twice daily), which was well tolerated. The laboratory tests revealed a complete remission. Hemoglobin and platelets were in the normal range.

## Discussion

A similar case of a patient with neuroborreliosis and detection of clonal B lymphocytes without previously known CLL has been described in the literature (8). After remission of the meningitis symptoms and reduction of cell count in CSF post antimicrobial and systemic chlorambucil therapy, leukemic cells were still detected in CSF in the same level. Their elimination was only finally achieved by intrathecal treatment with dexamethasone. It was presumed that the neuroborreliosis allowed the transmigration of leukocytes, including leukemic lymphocytes, across the blood-brain barrier. Matrix metalloproteinase 9 may contribute to open the blood-brain barrier in meningitis. A review article summarized the pathophysiology of the blood-brain barrier in infection and inflammation (9), including changes in IL-1β/prostaglandin E2 signaling, enhanced cellular trafficking, solute permeability and occasional direct damage to the endothelium. Increased blood-brain barrier permeability with age has already been confirmed, while aged septic rats showed more profound blood-brain barrier impairment affecting both solute and neutrophil reflux (10). Blood-brain barrier responses may occur with a delay after the onset of the systemic inflammation. Sustained inflammation is more likely to overcome perivascular microglial protective capacity, allowing innate immune cell infiltration (11). In our case, permeability or impairment of blood-brain barrier could not be excluded, since regional IgM synthesis was slightly higher than in serum.

Another case of a 75-year-old male patient, who was initially suspected for CLL leptomeningeal involvement, was eventually diagnosed as neuroborreliosis after careful immunophenotyping of the CSF samples and evaluation for infectious causes of lymphocytic meningitis (12). A careful differential diagnosis was also performed in our case and leukemic infiltration at initial stage of disease was ruled out using multiple diagnostic assays. It should be noted, lymphocytic meningitis in CLL may tell a leukemic meningeal involvement, only when CSF cells show a predominant immunophenotype of CLL cells and when any infectious cause, especially neurologic Lyme disease, has been excluded. But a post-infectious leptomeningeal involvement in CLL should not be underestimated.

Intriguingly, leptomeningeal involvement in our case occurred after the effective treatment of CNS infection in a patient with untreated, low-risk CLL (Binet stage A). Despite clinical progression with CNS involvement, the molecular and cytogenetic profile remained low risk. It is not an isolated case but uncommon. An international study conducted by European Research Initiative on CLL (ERIC) has recently reported that only 20% of CLL patients at diagnosis of CNS involvement had Binet A, meanwhile more patients possessed adverse prognostic markers, such as 75,9% with unmutated IGHV and 65% with chromosomal abnormalities (13). Considering the case of our patient and previous reports, the question arises whether the detection of leukemic cells in the CSF of CLL patients with/after infectious meningitis (especially in neuroborreliosis) is an extramedullary manifestation or a transmigration of clonal lymphocytes caused by the inflammation of the blood-brain barrier.

There is currently no consensus on the strategy to treat CNS involvement in CLL, which is physiologically significantly distinct from Richter’s transformation/primary CNS lymphoma. A conventional treatment is intrathecal chemotherapy. Intrathecal injections may contribute to a rapid anti-tumor effect on leptomeningeal involvement of hematological malignancies, especially in the early treatment phase (14). The most common regimen is classic triple therapy of cytarabine, methotrexate and corticosteroids (15). Nevertheless, in a case of optic neuropathy by CLL with leptomeningeal involvement, four weekly doses of intrathecal methotrexate mono and chlorambucil orally for 6 days have also induced sustained visual recovery (16). Furthermore, compared with methotrexate mono, intrathecal triple therapy may decrease CNS relapse but seems to be associated with increased incidence of bone marrow and testicular relapse in standard-risk acute lymphoblastic leukemia (17). In clinical practice, intrathecal chemotherapy may be the most appropriate first-line therapy for CNS involvement of indolent lymphomas, particularly in the presence of CNS infections, which require more differential diagnostic assays and careful assessment for medicine options and initiation of systemic anti-tumor therapy.

Although the current stand of care for aggressive lymphomas with CNS manifestations is high-dose methotrexate-based induction followed by thiotepa-containing conditioning and autologous stem cell transplantation (18), this approach is barely used on CLL patients. Before entering the era of targeted therapy for CLL, chemotherapy such as cyclophosphamide, fludarabine and methotrexate were most commonly used for CNS manifestations of CLL because of good blood-brain barrier penetration (19). Thus, in cases where patients are not fit for intrathecal chemotherapy or other alternatives, chemotherapy with a well-established CSF penetration regimen may still be indicated. Since Ofatumumab was approved by Food and Drug Administration (FDA) in 2009, anti-CD20 monoclonal antibodies are increasingly used for CLL treatment (20). At present, ESMO (21) and NCCN guidelines (22) recommend obinutuzumab for patients with previously untreated CLL and rituximab for relapsed/refractory disease. These anti-CD20 monoclonal antibodies are generally used in combination with Bcl-2 inhibitor or BTKi. Rituximab has already proven its efficacy in the CNS in both aggressive (23) and indolent lymphomas (24). Venetoclax as a selective BCL-2 inhibitor is an important component of targeted therapy for CLL. Some case reports have shown CNS efficacy of venetoclax, either in combination with intrathecal chemotherapy (25), or with high-dose methotrexate and rituximab (26). BTKi not only play a central role in CLL treatment overall (27), but have also repeatedly demonstrated the efficacy in CNS manifestations. Ibrutinib, which revolutionized the treatment of B-cell lymphoma, achieved durable effect in an Asian CLL patient with CNS involvement at the initial diagnosis (28). In 2024, Mayo Clinic reported a similar case with partial clinical and radiologic response to Zanubrutinib, but developing gastrointestinal hemorrhage (29). Unlike other reported cases of CLL with CNS involvement, this case describes subacute-onset encephalopathy in an untreated CLL patient with minimal symptoms, but lacks risk profiling. In addition, many studies of BTKi treatment for primary CNS lymphoma have also yielded encouraging results (30). Ibrutinib and Zanubrutinib are listed in the NCCN guidelines for recurrent/refractory primary CNS lymphoma (31). In our case, Zanubrutinib was used to treat leptomeningeal involvement of a pre-existing CLL after CNS infections. Given the advanced age of our patient, the following considerations were taken into account when selecting medication. To reduce the risk of tumor lysis syndrome, we excluded BCL-2 inhibitors and selected BTKi (32). Many studies have confirmed that Ibrutinib treatment increases the risk of developing cardiovascular diseases through interacting with off-BTK target proteins and inhibiting various cardiac signaling pathways (33), therefore, Zanubrutinib was used in this case. Our patient has been neurologically symptom-free for more than 18 months to date with ongoing but reduced dose of Zanubrutinib treatment.

The limitation in our case is that we did not perform any further molecular and immunological tests, such as NOTCH1 tests or the identification of stereotype subgroups. The risk profile was based on current conventional factors. Follow-up is ongoing.

## Conclusions

More attention should be paid to prophylaxis against CNS infections, meanwhile the potential association between such infections and the progression of CLL with CNS involvement warrants careful consideration. The optimal timing for initiation of anti-tumor therapy and management of leptomeningeal involvement in CLL remains uncertain, requiring individualized therapeutic approaches. Our case further supports the efficacy of BTK inhibitors.

## Data Availability

The original contributions presented in the study are included in the article/supplementary material. Further inquiries can be directed to the corresponding authors.
